# cAMP Response Element Binding Protein 1 (CREB1) Promotes Monounsaturated Fatty Acid Synthesis and Triacylglycerol Accumulation in Goat Mammary Epithelial Cells

**DOI:** 10.3390/ani10101871

**Published:** 2020-10-14

**Authors:** Dawei Yao, Chunlei Yang, Jing Ma, Lili Chen, Jun Luo, Yi Ma, Juan. J. Loor

**Affiliations:** 1 Tianjin Institute of Animal Husbandry and Veterinary Medicine, Tianjin Academy of Agricultural Sciences, Tianjin 300381, China; daweiyao1@yahoo.com (D.Y.); yangchunlei86@126.com (C.Y.); 15613563512@163.com (J.M.); chenlili0609@163.com (L.C.); 2 Shaanxi Key Laboratory of Molecular Biology for Agriculture, College of Animal Science and Technology, Northwest A&F University, Yangling, Xianyang 712100, Shaanxi, China; luojun@nwsuaf.edu.cn; 3 Mammalian NutriPhysioGenomics, Department of Animal Sciences and Division of Nutritional Sciences, University of Illinois, Urbana, IL 61801, USA

**Keywords:** fatty acid composition, monounsaturated fatty acids, goat milk, dairy nutrition

## Abstract

**Simple Summary:**

In non-ruminant liver and adipose tissue, cAMP response element binding protein 1(CREB1) is essential for lipid synthesis and triacylglycerol accumulation. The present study aimed to ascertain the role of CREB1 in regulating milk fatty acid composition synthesized by goat mammary gland. Our data found that overexpression of *CREB1* in vitro alters the abundance of lipogenic genes, triacylglycerol accumulation and concentration of monounsaturated fatty acids in goat mammary epithelial cells. Thus, manipulation of CREB1 in vivo might be one approach to improve the quality of goat milk.

**Abstract:**

cAMP response element binding protein 1 (CREB1) is a member of the leucine zipper transcription factor family of DNA binding proteins. Although studies in non-ruminants have demonstrated a crucial role of CREB1 in lipid synthesis in liver and adipose tissue, it is unknown if this transcription regulator exerts control of fatty acid synthesis in ruminant mammary cells. To address this question, we first defined the expression dynamics of *CREB1* in mammary tissue during lactation. Analysis of *CREB1* in mammary tissue revealed higher mRNA abundance in mammary tissue harvested at peak lactation. Overexpression of *CREB1* markedly upregulated sterol regulatory element binding transcription factor 1 (*SREBP1*), fatty acid synthase (*FASN*), acetyl-coenzyme A carboxylase α (*ACACA*), elongase of very long chain fatty acids 6 (*ELOVL6*), lipoprotein lipase (*LPL*), fatty acid binding protein 3 (*FABP3*), lipin 1 (*LPIN1*) and diacylglycerol acyltransferase 1 (*DGAT1*), but had no effect on glycerol-3-phosphate acyltransferase, mitochondrial (*GPAM*) or 1-acylglycerol-3-phosphate O-acyltransferase 6 (*AGPAT6*). In addition, overexpressing CREB1 led to a significant increase in the concentration and desaturation index of C16:1 (palmitoleic acid) and C18:1 (oleic acid), along with increased concentration of triacylglycerol. Taken together, these results highlight an important role of CREB1 in regulating lipid synthesis in goat mammary epithelial cells. Thus, manipulation of CREB1 in vivo might be one approach to improve the quality of goat milk.

## 1. Introduction

Compared with cow milk, goat milk contains a much greater proportion of monounsaturated fatty acids (MUFA) which enhance nutritional value in terms of human health including benefits for control of cardiovascular conditions and hypercholesterolemia or hyperlipemia [1]. Both stearoyl coenzyme A desaturase 1 (SCD1) and elongase of very long chain fatty acids 6 (ELOVL6) are critical regulators of MUFA synthesis [2,3,4], with the main products of the reactions being palmitoleic acid (PAM, 16:1n-7) or oleic acid (18:1n-9) [5,6,7]. These MUFA are the main substrates for synthesis of triacylglycerol (TAG) and cholesterol ester (CE) in milk.

The protein CREB is a ubiquitous transcription factor that has already been reported to play a role in the control of memory [8], cell proliferation [9] and differentiation [10]. Several lines of evidence implicate CREB in the regulation of cellular lipid metabolism [11,12]. More recent work in rodents using *CREB* ectopic expression demonstrated that it is strongly associated with TAG concentration. In *CREB* (-/-) mouse embryonic fibroblasts, the accumulation of cytoplasmic TAG was markedly reduced compared with the wild type group [13]. More powerful evidence for CREB in the control of TAG metabolism was obtained via *CREB* knockdown with specific antisense oligonucleotides. Results revealed that it not only decreased TAG concentrations in mouse liver, but also affected the expression of several genes involved in synthesis of fatty acids (FA) and TAG, including acetyl-coenzyme A carboxylase alpha 1 (*ACACA1*), *SCD1* and diacylglycerol acytransferase 2 (*DGAT1*) [14]. In addition, the CREB protein-associated coactivator CREB binding protein (CBP) enhanced the transcriptional activity of sterol regulatory factor binding protein (SREBP) [15]. SREBP1 plays an important role in regulating the synthesis of fatty acid and TAG in goat mammary cells [16]. Collectively, based on non-ruminant data, it appears likely that CREB exerts some degree of control over fatty acid synthesis in tissues such as ruminant mammary gland.

Compared with rodents, the signaling pathways involved and the role of CREB1 in ruminant mammary cells are not well known. Therefore, this study attempted to examine the extent to which overexpression of *CREB1* in goat mammary epithelial cells could induce changes in abundance of a series of key lipogenic target genes as well as TAG synthesis. Furthermore, we used fatty acid analysis to determine to which extent CREB1 could alter the content of monounsaturated fatty acids known to originate from de novo synthesis.

## 2. Materials and Methods

### 2.1. Animals and Goat Mammary Gland Tissue Collection

Three-year-old healthy Xinong Saanen dairy goats from the Northwest A&F University experimental farm (Yangling, Shaanxi, China) were used in this study. All animal collection and protocols were approved by the Animal Care and Use Committee of the Northwest A&F University. Mammary tissue samples harvested via biopsy were collected during peak lactation (60 d postpartum) and the dry period (60 d before expected parturition) (six goats were collected for each period). Percutaneous biopsies were performed from the right udder according to methods published previously [17]. All tissue samples were washed with diethylpyrocarbonate (DEPC)-treated water (Sigma, St. Louis, MO, USA) under sterile conditions within 20 min prior to storage in liquid nitrogen until RNA extraction.

### 2.2. Cloning of the CDS Region of CREB1 and Bioinformatics Analysis

The sequence of the CDS region of *CREB1* (Genbank accession: MK158073.1) was amplified from cDNA of goat mammary gland. Primers were designed based on information from the Yunnan black goat genome. The primers are as follows: forward, 5′-GAGAAGCGGAGTGTTGGT-3′; reverse, 5′-AGGTGTTGGAGCATTCCACAG-3′. The sequence was amplified using PrimeSTAR HS DNA Polymerase (Takara Bio Inc, Otsu, Japan). Amplified products were sequenced by Shenggong (Beijing, China). The details of the PCR and cloning procedure are in the Supplementary Materials.

The homology of nucleotide and amino acid sequences of the dairy goat CREB gene was compared to that of cow (Bos taurus, AF006042.1), human (Homo sapiens, BC095407.1) and mouse (Mus musculus, U46027.1) using Blastn (http://www.ncbi.nlm.nih.gov/BLAST/; Bethesda; Rockville, MD, USA).

### 2.3. Generation of Adenovirus

The cDNA sequence of dairy goat *CREB1* (Gene ID: MK158073.1) was subcloned into the pAdTrack-CMV shuttle vector between the Kpn I and Hind Ш (New England BioLabs Inc., Ipswich, MA, USA) restriction sites to generate the pAdTrack-CMV-CREB1 vector. Subsequently, the shuttle vector was linearized with the restriction endonuclease Pme I (New England BioLabs Inc., Ipswich, MA, USA) and inserted into Escherichia coli BJ5183 cells containing the backbone vector (pAdEasy-1). The linearized adenoviral plasmid by Pac I was transfected into 293A cells to pack the adenovirus containing cAMP response element binding protein 1 (Ad-CREB1) using a commercial system (AdEasy, Stratagene, La Jolla, CA, USA) as published by our group [18]. The recombinant adenovirus containing green fluorescent protein (GFP) was used as a control (Ad-GFP) and was a gift from Zhijie Chang (Tsinghua University, Beijing, China).

### 2.4. Cell Culture and Treatment

Goat primary cells were isolated from peak-lactation mammary tissue samples from Xinong Saanen goats and were cultured as described previously by our group [19]. Briefly, cells were cultured in an environment of 5% CO_2_ in air at 37 °C. Goat mammary epithelial cells were cultured in basal DF-12 (DMEM/F12; Hyclone, Logan, UT, USA) medium containing 5 μg/mL insulin (Sigma-Aldrich, St. Louis, MO, USA), 1 μg/mL hydrocortisone (Sigma-Aldrich), 10 ng/mL epidermal growth factor 1 (EGF-1, Invitrogen), 10% fetal bovine serum (Hyclone) and penicillin/streptomycin (100 U/mL Harbin Phamaceutical Group Co. Ltd., Harbin, China), and the culture medium was changed every 24 h. Before the beginning of experiments, goat mammary epithelial cells (GMEC) were cultured in lactogenic medium containing prolactin (2 μg/mL; Sigma-Aldrich) for 48 h [18]. The GMEC were plated before infection in 24-well plates and then infected with Ad-GFP or Ad-CREB1 recombinant adenovirus when cells reached approximately 80 to 90% confluence. The transfected GMEC were collected after 48-h incubation with adenovirus and then for lipid extraction, total RNA extraction, and TAG assay. The experiment was repeated 3 times.

### 2.5. RNA Extraction and Quantitative Real-Time PCR

Total RNA was extracted from tissues and GMEC using Trizol reagent (Invitrogen Corp., Carlsbad, CA, USA) following the manufacturer’s instructions. The quality of RNA was examined by agarose gel electrophoresis analysis of the integrity of 28S and 18S rRNA. The cDNA was synthesized from 0.5 μg of purified RNA using a PrimeScript RT Kit with gDNA Eraser (Perfect Real time, Takara Bio Inc., Otsu, Japan) to remove genomic DNA contamination. Quantitative real-time PCR (qPCR) to determine the relative expression of target genes in tissue and GMEC was performed according to a previous study [19]. Data were normalized to ubiquitously expressed transcript (UXT), mitochondrial ribosomal protein L39 (MRPL39) and ribosomal protein S9 (RPS9) [20]. Every sample was analyzed in triplicate. Quantitative real-time PCR (qPCR) primer sequences are listed in Table 1.

### 2.6. Total Fatty Acid Extraction and Analysis

Prior to fatty acid extraction, goat mammary epithelial cells were washed 3 times with phosphate-buffered saline (PBS, Solarbio, Beijing, China) as described previously [6]. Total fatty acids were extracted and methylated according to a previous report from our laboratory [21]. Methylated samples were analyzed by gas chromatography equipment (GC) (Agilent, Santa Clara, CA, USA) using an HP-5 column. The relative proportions of FA were calculated as the percentages of the total peak areas that could be identified [25].

### 2.7. Measurement of Total Cellular TAG

Goat mammary epithelial cells were washed twice with cold PBS after 48 h of incubation with adenovirus. Intracellular TAG was measured using a TAG assay kit according to the manufacturer’s instructions (Applygen Technologies Inc., Beijing, China). Data were read on a microtiter plate reader (Bio-Rad Laboratories Inc., Berkeley, CA, USA). Protein concentrations were quantified using the BCATM protein assay kit (Thermo Fisher Scientific, Waltham, MA, USA). The TAG content was calculated by normalizing to total cellular protein abundance and reported as µg/mg protein.

### 2.8. Statistical Analysis

Results were analyzed with SPSS 19.0 (SPSS, Inc., Chicago, IL, USA) and the data are presented as means ± standard error of the means (SEM) from each of three independent experiments. Data of qPCR were analyzed using the 2−ΔΔCt method relative to the control group. The content of each identified fatty acid was calculated as a proportion of the total fatty acids, and the desaturation index was evaluated as the ratio between the desaturation product and the sum of the product and substrate [26]. Statistical analysis was performed using Student’s t-test. Differences between two treatments were considered statistically significant at *p* < 0.05.

## 3. Results

### 3.1. Cloning and Bioinformatics Analysis of the CREB1 Gene CDS Region

**Figure 1:**
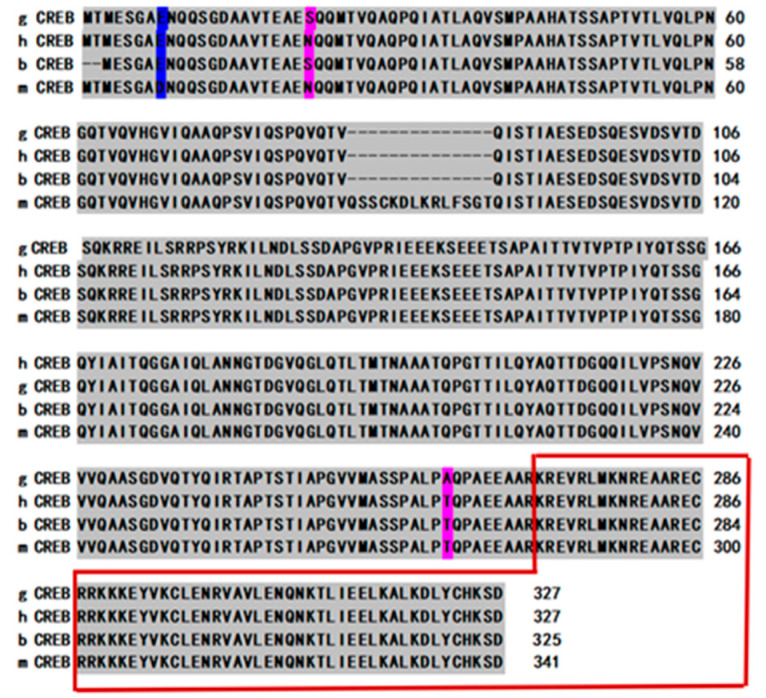
Comparison of amino acid sequences between dairy goat, human, cattle and mouse *CREB* gene. The gray portion indicates the aligned amino acids, the blue portion indicates a conservative substitution in the aligned amino acids, the pink portion indicates a nonconservative substitution in the aligned amino acids and the conservative representative bZIP regions are in the red line.

The total length of CDS was 984 bp, encoding 327 amino acids. The degree of similarity of the nucleotide sequences in the CDS region of goat *CREB1* compared with cow (AF006042.1), human (BC095407.1) and mouse (U46027.1) was 98%, 95% and 95%, respectively. The degree of similarity of amino acid sequences was above 95% for all species (Figure 1).

### 3.2. Expression Patterns of CREB1 in Different Lactation Stages in Dairy Goat

**Figure 2:**
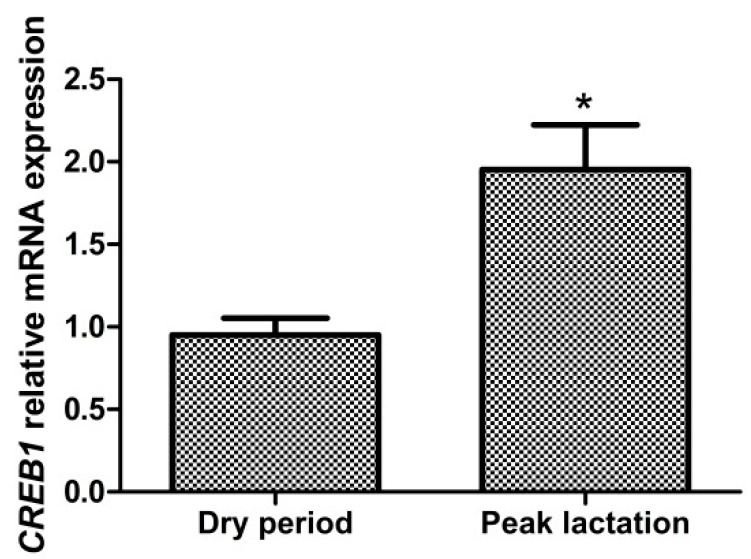
mRNA expression of cAMP response element binding protein 1 (*CREB1*) in mammary tissue from the dry period and peak lactation. Values are means ± SEM for 6 individuals. * Significantly higher (*p* < 0.05) compared with the dry period.

Compared with the dry period, expression of *CREB1* increased significantly (*p* < 0.05) at peak lactation (Figure 2). This result indicated CREB1 may play a role in lipid metabolism during lactation in goat mammary gland.

### 3.3. CREB1 Overexpression Influences Expression of Genes Related to Lipogenesis in GMEC

**Figure 3:**
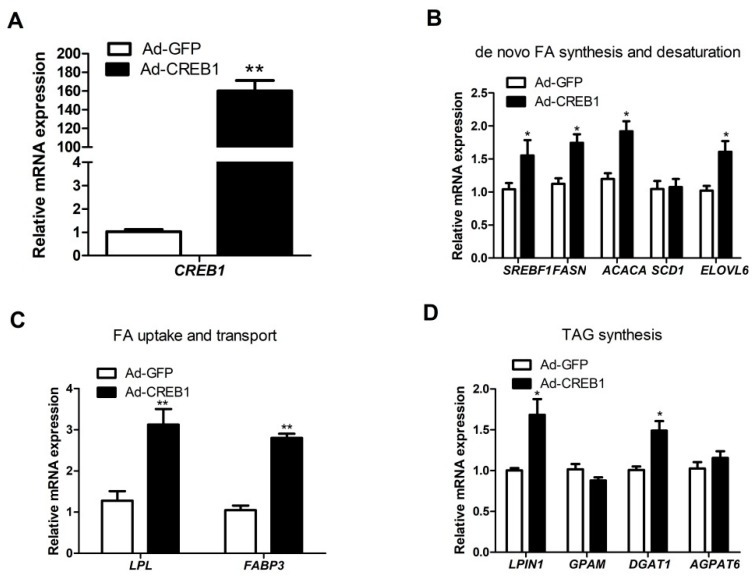
cAMP response element binding protein 1 (*CREB1*) influences the expression of genes related to fatty acid (FA) metabolism in goat mammary epithelial cells (GMEC). GMEC were transfected with Ad-CREB1 or Ad-GFP for 48 h and then collected for mRNA extraction. Panel (**A**). mRNA expression of *CREB1*. Panel (**B**). mRNA expression of genes related to de novo fatty acid synthesis and desaturation (*ACACA*, *FASN*, *SREBF1*, *SCD1*, *ELOVL6*). Panel (**C**). mRNA expression of genes related to FA uptake and transport (*LPL* and *FABP3*). Panel (**D**). mRNA expression of genes related to TAG synthesis and lipid droplet formation (*DGAT1*, *GPAM*, *LPIN1*, *AGPAT6*). Data are means ± SEM for three independent experiments. * *p* < 0.05 vs. control (Ad-GFP). ** *p <* 0.01 vs. control (Ad-GFP).

To explore the role of CREB1 in lipid metabolism, we generated recombinant adenovirus and detected the expression of genes related to lipogenesis in goat mammary epithelial cells. After cells were infected with Ad-CREB1 for 48 h, expression of *CREB1* increased >150-fold compared with controls (*p* < 0.01; Figure 3A). Overexpression of *CREB1* significantly upregulated the mRNA expression of *SREBF1* (*p <* 0.05), *FASN* (*p* < 0.05), *ACACA* (*p <* 0.05) and *ELOVL6* (*p <* 0.05) (Figure 3B). Overexpression of *CREB1* markedly increased transcription of genes involved in FA uptake and transport including *LPL* (*p <* 0.01) and *FABP3* (*p <* 0.01; Figure 3C). In addition, overexpression of *CREB1* increased *LPIN1* (*p <* 0.05) and *DGAT1* (*p <* 0.05), which are related to TAG synthesis, but had no effect on *GPAM* and *AGPAT6* (Figure 3D). Collectively, these data suggested that overexpression of CREB1 promoted lipid synthesis in GMEC.

### 3.4. CREB1 Overexpression Alters Content of Fatty Acids

**Figure 4:**
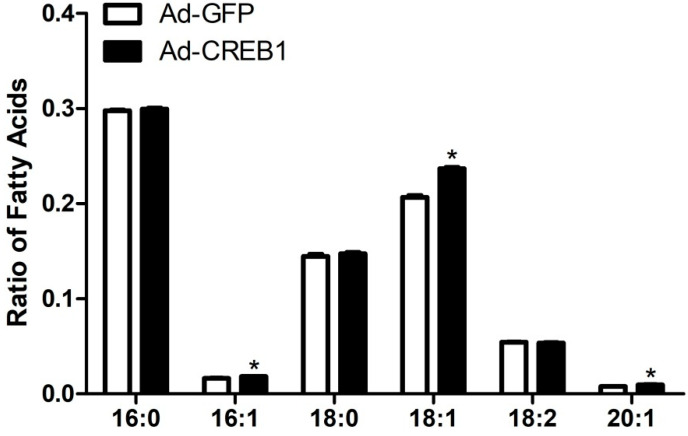
Overexpression of cAMP response element binding protein 1 (*CREB1*) changes intracellular fatty acid content. Data are means ± SEM for 3 individual experiments. * *p* < 0.05 vs. control (Ad-GFP).

In order to further investigate the effect of CREB1 on fatty acid synthesis in GMEC, the relative content of fatty acid was measured. CREB1 overexpression led to an increase in the proportion of C16:1 (palmitoleic acid), C18:1 (oleic acid) and C20:1 (eicosenoic acid), but had no marked effect on the proportion of C16:0 (palmitic acid), C18:0 (stearic acid) and C18:2 (octadecadienoic acid) (Figure 4). This result indicated that CREB1 may promote the synthesis of unsaturated fatty acids in GMEC.

### 3.5. CREB1 Overexpression Upregulates the Desaturation Index of C16:1, C18:1 and TAG

**Figure 5:**
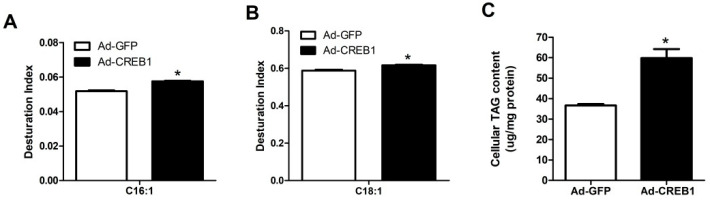
Overexpression of cAMP response element binding protein 1 (*CREB1*) changes the intracellular fatty acid (FA) desaturation index and triacylglycerol (TAG) content in goat mammary epithelial cells (GMEC). GMEC were transfected with Ad-CREB1 or Ad-GFP for 48 h and then collected for fatty acid analysis. Panel (**A**–**C**) depicted the C16:1 desaturation index, C18:1 desaturation index and total TAG, respectively, in GMEC infected with Ad-CREB1 compared with the control (Ad-GFP). Data are means ± SEM for 3 individual experiments. * *p* < 0.05 vs. control (Ad-GFP). Significance was declared at *p* < 0.05.

After GMEC were cultured with a virus for 48 h, the desaturation index of C18:1 and C16:1 was significantly upregulated (*p* < 0.05; Figure 5A,B). Furthermore, after overexpression of *CREB1*, intracellular TAG content increased significantly compared with the control group (*p* < 0.05; Figure 5C). These data implied that CREB1 may alter some of the dynamic mechanisms of lipid synthesis in goat mammary gland during lactation.

## 4. Discussion

The protein CREB is located primarily in the nucleus and plays a crucial role in initiating adipogenesis [27]. However, the role of CREB in lactating mammary gland has not been fully addressed. The present studies confirmed that the abundance of the CREB1 gene in mammary tissue is associated with lactation. Thus, combined with research in rodent liver or adipose tissue [11,14], this indicated that CREB is likely an important regulatory factor in lipogenic tissues such as lactating ruminant mammary gland.

Fatty acid metabolism in mammary epithelial cells includes the uptake and transport of exogenous fatty acid, de novo synthesis and desaturation and synthesis and secretion of TAG [20]. The process of de novo lipogenesis in mammary gland is strongly correlated with multiple enzymes including the rate-limiting enzyme ACACA and FASN. Our data revealed that expression of *ACACA* and *FASN* was markedly increased by *CREB1* overexpression, which is similar to results in 3T3-L1 cells [11]. Accumulating evidence has suggested that CREB can bind to predicted cAMP response elements (CREs) in the promoters of several adipogenesis-specific gene promoters, e.g., *SCD* and *FASN* [11,28]. CREB is also involved in the processing and maturation of SREBP, which directly regulates expression of *FASN* and *SCD1* [29,30]. Thus, we speculate that CREB participates in the transcription regulation of genes in two ways: one is to directly regulate transcription level of the target gene by binding to the CRE site in the promoter region of the target gene; another is to indirectly regulate transcription of the target genes by forming a co-activated transcription complex to activate the expression of SREBP. Peroxisome proliferator-activated receptor γ(PPARγ) is another pivotal transcription factor involved in the regulation of lipid synthesis in goat mammary gland [18]. In rodents, CREB controls hepatic lipid metabolism through inhibition of *PPARγ* [12]. *SCD1* is also directly regulated by PPARγ at the transcriptional level [30]. Thus, fatty acid-related genes are not only regulated by one transcription factor but may work through a regulatory network, of which may lead to the modest changes of the fatty acid-related genes upon *CREB* overexpression. Therefore, CREB is likely to have a positive effect on the synthesis of FA in a direct and indirect manner.

Besides the importance of de novo synthesis to generate an FA pool for synthesis of TAG, uptake of long-chain FA from peripheral circulation and intracellular transport are important in the context of milk fat synthesis [20]. Studies have shown that long-chain fatty acids coated with chylous granules in the blood are lipolyzed and released by LPL, followed by intracellular transport inside the cell via FABP3 prior to esterification into TAG synthesis. Expression of *FABP3* was the highest in the FABP family, and upregulation of *FABP3* in cow mammary gland during lactation was up to 80 times higher compared with the dry period [31]. Thus, the upregulation of *LPL* and *FABP3* due to overexpression of *CREB1* suggested that it can also help control exogenous uptake and intracellular channeling of FA. The promoter sequence of *FABP3* has a CREB binding site and is highly conserved in cattle, sheep and pigs [32]. Taken together, these results indicate that CREB may play a role in lipogenesis in goat mammary gland as in non-ruminants.

Overexpression of *CREB1* upregulated abundance of *ELOVL6*, which catalyzes the elongation of C12-C16 saturated or monounsaturated fatty acids [5,7]. A direct control of *ELOVL6* by CREB is unclear, but murine experiments indicate that CREB might act through SREBP1 [2]. Such a relationship was confirmed in the present study because overexpression of *CREB1* not only upregulated *SREBF1* but also *ELOVL6*. Although direct control of CREB1 on *ELOVL6* cannot be discounted, the increased proportions of C16:1, C18:1 and C20:1 in GMEC due to *CREB1* overexpression suggested a direct relationship. The content of unsaturated FA is also an important substrate for the synthesis of TAG, especially C18:1n-9. However, CREB did not affect the expression of *SCD1*, which disagrees with results in 3T3-L1 preadipocytes [28], but was in line with liver data from *CREB* knockdown mice [14]. The increase in the desaturation index of C16:1 and C18:1 was surprising. We speculate that this apparent discrepancy may be due to a “complementary action” between SCD isotypes, a unique isoform in ruminants named SCD5 [33] and the joint effect of ELOVL6.

The significant increase in the abundance of *LPIN1* and *DGAT1*, along with the marked TAG accumulation in GMEC when *CREB1* was overexpressed, underscored its role in esterification. The LPIN1 gene, a member of the LPIN family, is a key regulator of adipocyte differentiation and lipid metabolism [34]. This protein is a phosphatase that generates diacylglycerol, the substrate for the last step in the esterification process [35]. In addition, LPIN1 directly interacts with nuclear peroxisome proliferator-activated receptor α (PPARα) and PPARα costimulatory factor 1α (PCG1α) as a transcriptional co-regulator to regulate the expression of genes involved in FA utilization and lipid synthesis [36,37,38]. The increase in TAG accumulation in our study was consistent with a previous finding in 3T3-L1 adipocytes [39] but disagrees with data from the liver of mice with the *CREB* knockdown that displayed a fatty liver phenotype [12]. An increase in de novo FA synthesis and desaturation is associated with TAG accumulation in GMEC [1]. In fact, oleic acid (C18:1n-9) is the preferred substrate for synthesis of TAG [7]. To sum up, the results illustrate that CREB is required for TAG accumulation in lactating mammary gland.

Although the present study provided direct evidence that overexpression of *CREB1* enhances de novo fatty acid synthesis and lipid accumulation in GMEC, some limitations of the study need to be considered. First, the protein abundance of the lipogenic target genes needs to be determined to ascertain the response in a more quantitative fashion. Second, activity of some of these enzymes should be measured to better reflect and quantify the rate of fatty acid synthesis. Lastly, specific mechanisms for how the CREB protein binds to the CRE acting element and regulates its downstream lipogenic target genes need to be confirmed.

## 5. Conclusions

Upregulation of *CREB1* in lactating mammary tissue illustrated that it plays an important role in goat mammary gland. Similarly, upregulation of lipogenic genes, unsaturated fatty acid content and TAG accumulation demonstrated a direct role for CREB1 in modulating these processes. Thus, manipulating the expression of *CREB1* in vivo may serve as an important strategy to improve the quality of goat milk.

## Figures and Tables

**Table 1:** Characteristics of primers used and efficiency of the RT-qPCR reaction.

Gene1	Accession No.	Primer Sequences (5′-3′)	Source
*ACACA*	XM_005693156.1	F: CTCCAACCTCAACCACTACGG	[21]
		R: GGGGAATCACAGAAGCAGCC	
*AGPAT6*	NM_001083669.1	F: AAGCAAGTTGCCCATCCTCA	[21]
		R: AAACTGTGGCTCCAATTTCGA	
*CREB1*	MK158073.1	F: CACTCAGCCAGGCACTACCA	This manuscript
		R: GGAAGACGCCATAACAACCC	
*DGAT1*	XM_005688895.1	F: CCACTGGGACCTGAGGTGTC	[21]
		R: GCATCACCACACACCAATTCA	
*ELOVL6*	NM_001102155.1	F: GGAAGCCTTTAGTGCTCTGGTC	[22]
		R: ATTGTATCTCCTAGTTCGGGTGC	
*FABP3*	NM_001285701.1	F: GATGAGACCACGGCAGATG	[21]
		R: GTCAACTATTTCCCGCACAAG	
*FASN*	DQ915966.3	F: GGGCTCCACCACCGTGTTCCA	[21]
		R: GCTCTGCTGGGCCTGCAGCTG	
*GPAM*	AY515690	F: ATTGACCCTTGGCACGATAG	[21]
		R: GATGAGACCACGGCAGATG	
*LPL*	DQ997818	F: AGGACACTTGCCACCTCATTC	[21]
		R: TTGGAGTCTGGTTCCCTCTTGTA	
*LPIN1*	NM_002707716	F: TCCCTGCTCGGACGTAATTG	[23]
		R: TGGCCACCAGAATAAAGCATG	
*MRPL39*	XM_005674737.1	F: AGGTTCTCTTTTGTTGGCATCC	[24]
		R: TTGGTCAGAGCCCCAGAAGT	
*RPS9*	DT860044	F: CCTCGACCAAGAGCTGAAG	[24]
		R: CCTCCAGACCTCACGTTTGTTC	
*SCD1*	GU947654	F: CCATCGCCTGTGGAGTCAC	[21]
		R: GTCGGATAAATCTAGCGTAGCA	
*SREBF1*	HM443643.1	F: ACGCCATCGAGAAACGCTAC	[21]
		R: GTGCGCAGACTCAGGTTCTC	
*UXT*	XM_005700842.1	F: TGTGGCCCTTGGATATGGTT	[24]
		R: GGTTGTCGCTGAGCTCTGTG	

Annealing temperature for all primers in this table is 60 °C. F, forward primer; R, reverse primer. *ACACA*, acetyl-coenzyme A carboxylase α; *AGPAT6*, 1-acylglycerol-3-phosphate O-acyltransferase 6; *CREB1*, cAMP response element binding protein 1; *DGAT1*, diacylglycerol acyltransferase 1; *ELOVL6*, elongase of very long chain fatty acids 6; *FABP3*, heart-fatty acid binding protein 3; *FASN*, fatty acid synthase; *GPAM*, glycerol-3-phosphate acyltransferase; *LPL*, lipoprotein lipase; *LPIN1*, lipin 1; *MRPL39*, mitochondrial ribosomal protein L39; *RPS9*, ribosomal protein S9; *SCD1*, stearoyl-coenzyme A desaturase 1; *SREBF1*, sterol regulatory element binding transcription factor 1; *UXT*, ubiquitously expressed transcript.

## Supplementary Materials

The following are available online at https://www.mdpi.com/2076-2615/10/10/1871/s1, Figure S1: Cloning and Identification of the CDS region of goat cAMP response element binding protein 1 (*CREB1*).
