# Supplementary material for: cAMP Response Element Binding Protein 1 (CREB1) Promotes Monounsaturated Fatty Acid Synthesis and Triacylglycerol Accumulation in Goat Mammary Epithelial Cells
Source: Animals (Basel). 2020 Oct 14;10(10):1871. doi: 10.3390/ani10101871 (PMC7602241; doi:10.3390/ani10101871)
Supplement: Supplementary file 1 [file animals-10-01871-s001.pdf]

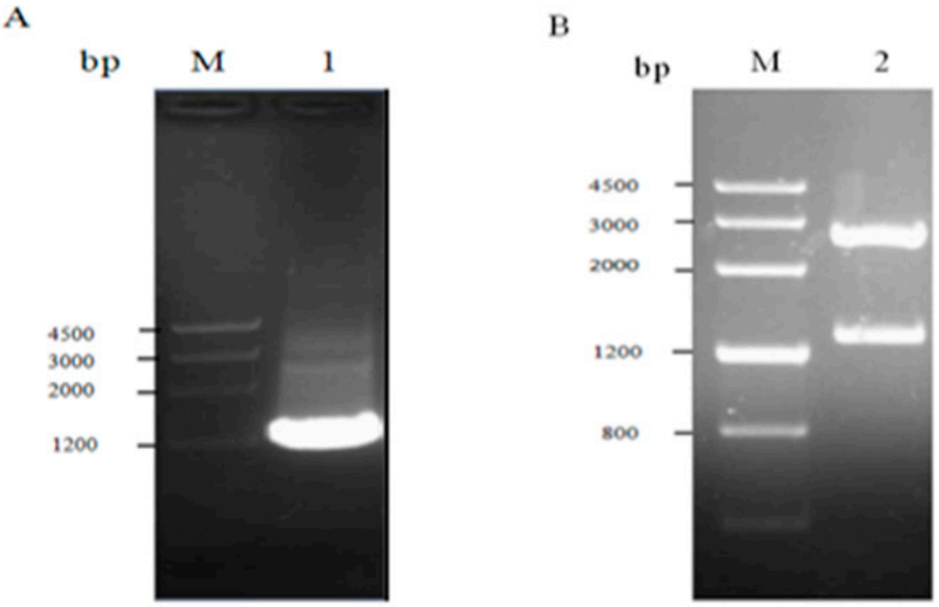

Figure S1. Cloning and Identification of the CDS region of goat cAMP response element binding protein 1 (*CREB1*). M. DNA marker III; (**A**). Amplification product of *CREB1* CDS region by PCR; (**B**). pMD19-T-CREB1 vector digested by *Sal* I and *EcoR* I.

PCR of the goat *CREB1* was performed in 25-μL reaction mixtures containing 1 μL of cDNA template using PrimeSTAR HS DNA Polymerase (Takara Bio Inc, Otsu, Japan). The PCR cycling conditions were performed according to the manufacturer's instructions. Amplified products were about 1200 bp as shown in Figure1 A and then cloned into the pMD19-T vector. The pMD19-T-CREB1 vector was then digested by *Sal* I and *EcoR* I (Takara, Japan). Two binds were shown in Figure1 B, one is the CDS region of *CREB1*, the other is the pMD19-T vector.
